# Orthogonal position representations for transformer in neural machine translation

**DOI:** 10.1371/journal.pone.0334443

**Published:** 2025-10-15

**Authors:** Yue Zhao, Qinghong Zhang, Shuhan Zhou

**Affiliations:** 1 School of Marxism, Northeastern University, Shenyang, China; 2 School of Computer Science and Engineering, Northeastern University, Shenyang, China; Industrial University of Ho Chi Minh City, VIET NAM

## Abstract

In recent years, the Transformer architecture has solidified its position as the dominant model in neural machine translation (NMT), thanks to its exceptional effectiveness in capturing long-range dependencies and remarkable scalability across diverse linguistic tasks. A key characteristic of the Transformer is its reliance on self-attention mechanisms, which, while powerful, are inherently position-insensitive—treating tokens as a set rather than an ordered sequence. This limitation makes positional encoding a critical component in Transformer-based models and their variants, as it provides the necessary sequential context to differentiate token positions within a sequence. In this paper, we address this challenge by proposing a novel orthogonal fixed-dimension positional representation (OPR). This design is meticulously engineered to maximize the discrimination of positions within a sequence, ensuring that each position is uniquely and distinctively encoded. Notably, OPR introduces no additional parameters to the model and incurs no extra computational overhead, making it highly efficient for real-world applications. Our experimental evaluations, conducted across multiple standard NMT datasets, demonstrate that OPR consistently outperforms several strong baselines, including traditional sine-cosine positional encoding and learnable positional embeddings. It achieves notable improvements in both BLEU and COMET scores, with gains observed across all tested language pairs. Furthermore, when combined with relative positional encoding (RPR), the OPR method’s performance is further enhanced, highlighting its ability to effectively model both absolute and relative positional relationships—a dual capability that is crucial for nuanced sequence understanding.

## 1 Introduction

In the field of neural machine translation (NMT), the Transformer model has emerged as the dominant framework, revolutionizing the landscape of language translation since its introduction by Vaswani et al. [1]. Unlike its predecessors such as Recurrent Neural Networks (RNNs) [2–4] and Convolutional Neural Networks (CNNs) [5], the Transformer’s encoder-decoder architecture, powered by self-attention mechanisms, enables efficient parallel computation and superior capture of long-range contextual dependencies—two critical advantages that have made it the de facto standard for state-of-the-art NMT systems [6,7]. Recent advancements in large language models (LLMs) have further solidified its foundational role, with variants like mT5 [8] and LLaMA-2 [9] demonstrating remarkable cross-lingual capabilities built upon Transformer backbones. Its widespread adoption across diverse language pairs and translation scenarios, from general domain tasks to specialized fields like technical documentation and cross-lingual dialogue, underscores its versatility and robustness. As research in NMT continues to advance, optimizing key components of the Transformer has become a focal point for enhancing translation quality, with positional encoding standing out as a critical element that directly influences the model’s ability to interpret sequence order [10].

Positional Encoding is a core component of the Transformer model [11–18]. In traditional Recurrent Neural Networks (RNNs) or Long Short-Term Memory Networks (LSTMs), the sequential order is implicitly captured through the network’s recursive structure [19,20]. However, the parallel computation structure of the Transformer lacks the explicit ability to model sequence order, and therefore, positional encoding is introduced to enable the model to understand the relative or absolute positions of words in the input sequence. The primary task of positional encoding is to assign a unique vector representation to each input position, which is then added to the input word embeddings, allowing the model to distinguish between different positions during computation. Recent studies have shown that suboptimal positional encoding can lead to performance degradation in low-resource language pairs [21], highlighting the need for more robust designs.

Despite the widely recognized importance of positional encoding, many existing positional encoding methods do not perform as expected in certain tasks. This is because, in many Natural Language Processing (NLP) tasks, sentence length and structure exhibit significant variability [1,22,23]. For example, long sentences may rarely appear in training data, making it difficult for the model to learn meaningful positional information from these sentences. Additionally, fixed-length word embeddings and positional encodings may lead to information loss or waste model capacity when handling variable-length inputs. Thus, designing more efficient positional encoding methods that can handle input sequences of varying lengths has become an urgent challenge, especially with the growing demand for translating longer documents in academic and professional settings [24].

To address this problem, researchers have proposed periodic positional encoding methods based on periodic functions [22,25–31]. Periodic functions naturally scale and can effectively handle variable-length input sequences. Classic periodic positional encoding methods, such as Sinusoidal Positional Encoding, generate position encodings using sine and cosine functions, providing smooth, continuous position representations for sequences of arbitrary lengths. Although this method has been widely adopted in Transformer models, it still faces challenges, particularly in terms of the lack of distinguishability of position encodings in high-dimensional spaces. Recent alternatives such as Rotary Position Embedding (RoPE) [15,32] enhance positional awareness by rotating query and key vectors to naturally encode relative positional relationships. However, this design introduces additional computational complexity due to the need for dimension-wise rotation operations on these vectors.

In this paper, we propose a novel method termed Orthogonal Position Representation (OPR). This approach enhances the distinguishability of position encodings, with a particular focus on positions that are far apart, thereby boosting the Transformer model’s capability to discern the positions of input elements and ultimately improving the translation quality of neural machine translation systems.Compared to traditional periodic positional encoding methods, OPR leverages the orthogonality inherent in sine and cosine functions—drawing on their property that the integral of products of distinct sine or cosine functions over the interval [0, 2π] equals zero—to design position encodings. This not only strengthens the distinguishability of position representations but also, by introducing a coefficient *k* to modulate the degree of orthogonality, captures the underlying relational information between neighboring positions to a certain extent. This balance is crucial for enhancing Transformer performance. Notably, unlike rotation-based approaches such as RoPE, OPR achieves these advantages without incurring additional computational complexity from vector rotation operations.

We validate the OPR method across several machine translation tasks and compare it with a range of strong baseline models. Experimental results show that OPR consistently achieves significant improvements in BLEU [33] and COMET [34] scores across various settings, demonstrating strong distinguishability and effective modeling of different positions. Notably, we observe that OPR effectively distinguishes position encodings when processing short sentences, while also mitigating the high-dimensional redundancy problem encountered by traditional positional encoding methods in longer sentences. Furthermore, OPR captures relative distance information between positions, which plays an essential role in enhancing the translation quality of machine translation models. By introducing orthogonality, OPR effectively addresses the issues of blurry positional information and underutilized model capacity inherent in traditional positional encoding methods.

## 2 Background

The Transformer [1] follows the standard encoder-decoder architecture. Both the encoder and decoder are composed of several stacked attention modules and feed-forward networks. Due to the inability [35] of the self-attention mechanism and the feed-forward network structure to capture word position information, the model requires a method to integrate word positions and their relative order into the Transformer model, known as the positional encoding method. Positional encoding methods can be mainly divided into two types: Absolute Positional Encoding (APE) [36] and Relative Positional Representation [11].

### 2.1 Absolute positional encoding

**Fig 1:**
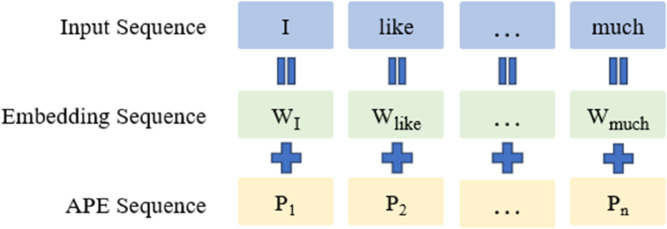
Absolute positional encoding method.

The Absolute Positional Encoding [11,13] method informs the Transformer model of the absolute position of each word in the input sentence, similar to assigning a “position label” to each element in the input sequence, indicating its absolute position in the sentence. Since the input to the Transformer model is a sequence of word embeddings, the simplest and most effective method of absolute positional encoding is to append the encoding of each word’s corresponding absolute position to its respective word embedding in the input sequence, as shown in Fig 1.

Mathematically, let the input word embedding sequence be denoted as $w=\{w_{1},w_{2},\ldots ,w_{n}\}$, where the sequence *w* is a matrix of size *n* × *d*, with *w*_*i*_ representing the word embedding at position *i*, *n* being the number of words in the sentence, and *d* being the dimensionality of the word embeddings. Through the absolute positional encoding method, we can obtain a positional encoding sequence of the same size as the word embedding sequence, denoted as $p=\{p_{1},p_{2},\ldots ,p_{n}\}$, where *p*_*i*_ represents the positional encoding at position *i*, with the same dimensionality *d*. The word embedding matrix is then added to the positional encoding matrix, resulting in the final input sequence to the model: $e=\{w_{1}+p_{1},w_{2}+p_{2},\ldots ,w_{n}+p_{n}\}$. The classic absolute positional encoding method can be divided into two types: trainable absolute positional encoding and sinusoidal absolute positional encoding. Here, we only introduce the sinusoidal absolute positional encoding method.

In the Transformer [1] model, the method of absolute position encoding using sine and cosine functions was proposed, as follows:

$$P\left(pos,2j\right)=\sin\left(pos\cdot c^{\frac{2j}{d}}\right)$$ (1)

$$P\left(pos,2j\;+\;1\right)=\cos\left(pos\cdot c^{\frac{2j}{d}}\right)$$ (2)

In this method, *P*(*pos*, 2*j*) represents the value at the even dimension 2*j* for the position pos in the sequence, and P(pos,2j+1) represents the value at the odd dimension 2*j* + 1 for the same position pos. Here, *d* is the dimensionality of the position encoding, and *c* is an empirical coefficient with a value of 1/10000.

### 2.2 Relative positional representation

Although the sine-cosine absolute position encoding method enables the Transformer model to explicitly model absolute positional information, it still cannot explicitly capture the relative distances between elements. Additionally, since the absolute position encoding typically operates at the input of the Transformer model, there is a risk of the absolute positional information being forgotten as the model depth increases. To address these issues, [11] introduced the concept of relative position representations as a supplement to the “absolute positional information.” The relative position encoding [18] method operates within the self-attention mechanism of each layer in both the encoder and decoder of the Transformer model, allowing the model to explicitly capture the relative positional information between elements.

[11] extended the dot-product self-attention mechanism by introducing relative positional information into the computation. When calculating the relevance between position *i* and position *j*, they simultaneously consider the positional relationship between *i* and *j*. Formally, the formula is improved as follows:

$$z_{i}=\sum_{j=1}^{n}a_{ij}(V_{j}+a_{j-i}^{V})$$ (3)

$$a_{ij}=\text{Softmax}\left(\frac{Q_{i}{\left(K_{j}+a_{j-i}^{K}\right)}^{T}}{\sqrt{d_{z}}}\right)$$ (4)

In this, $\alpha_{j-i}^{V}$ and $\alpha_{j-i}^{K}$ are the relative position embeddings at positions *i* and *j* on *K*_*j*_ and $V_{j}$, respectively, and are trainable parameters of the model. When the length distributions of the training and test data are inconsistent, there is a risk that some relative position embeddings may not be trained. To address this issue, the method sets a maximum threshold *k* for the relative distance, enhancing the model’s focus on the *k* words before and after the current word. When the absolute value of the relative distance exceeds *k*, $\alpha_{k}^{V}$ or $\alpha_{-k}^{V}$ is used uniformly instead. Therefore, the final window size for the relative positions is 2*k* + 1. The specific calculation formula is as follows:

$$a_{j-i}^{K}=w_{clip(j-i,k)}^{K}$$ (5)

$$a_{j-i}^{V}=w_{clip(j-i,k)}^{V}$$ (6)

clip(x,k)=max(−k,min(k,x)) (7)

**Fig 2:**
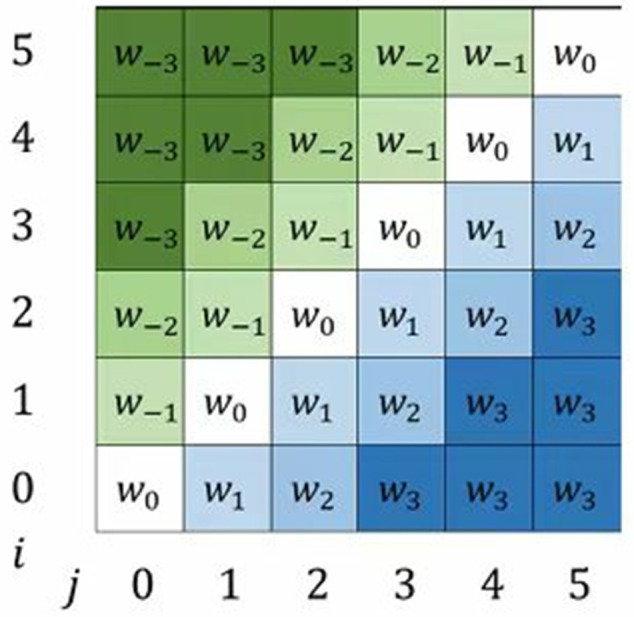
Relative positional embedding matrix.

The relative position embedding is embedded as shown in Fig 2. Specifically, the embedding at the *i*-th row and *j*-th column represents the relative position of the element at position *j* with respect to the element at position *i*, denoted as $w_{clip(j-i,k)}^{K}$. Additionally, this method trains independent relative position embeddings for each encoding and decoding layer in the Transformer model, which helps the model capture relative position information at different depths.

## 3 Methods

In this section, we introduce the Orthogonal Position Representation (OPR) method. This method is designed to enhance the distinguishability between position encodings of distant positions while retaining the sequence extrapolation capability critical for real-world NMT applications. By optimizing positional information modeling, this method directly strengthens the Transformer’s ability to discern the order of input elements, thereby mitigating errors caused by positional ambiguity and ultimately improving the translation quality of NMT systems.

### 3.1 Orthogonality of sine and cosine functions

This section formally proves the orthogonality of sine and cosine functions with distinct frequencies over the interval [0,2π]—a fundamental mathematical basis for OPR’s design.

For integers $m,n\in {\mathbb{N}}^{+}$ (where m≠n), two key orthogonality conditions hold, as detailed below:

**1. Orthogonality of sine functions.** We aim to prove:

$$\int_{0}^{2\pi }\sin(nx)\sin(mx)\;dx=0$$ (8)

Start with the trigonometric product-to-sum identity, which decomposes the product of sines into a difference of cosines:

$$\sin A\sin B=\frac{1}{2}\left[\cos(A-B)-\cos(A+B)\right]$$ (9)

Let *A* = *nx* and *B* = *mx* (corresponding to the frequency-related terms in positional encoding). Substituting these into the identity yields:

$$\sin(nx)\sin(mx)=\frac{1}{2}\left[\cos\left((n-m)x\right)-\cos\left((n+m)x\right)\right]$$ (10)

Integrate both sides over the interval [0,2π]. By the linearity of integration (the integral of a difference equals the difference of integrals), we split the expression into two separate integrals:

$$\int_{0}^{2\pi }\sin(nx)\sin(mx)\;dx=\frac{1}{2}\left(\int_{0}^{2\pi }\cos\left((n-m)x\right)dx-\int_{0}^{2\pi }\cos\left((n+m)x\right)dx\right)$$ (11)

For cosine integrals of the form ∫cos(kx)dx (where k≠0), the antiderivative is $\frac{1}{k}\sin(kx)+C$. Here: - Since m≠n, n−m≠0. Let *k*_1_ = *n*−*m*; evaluating the first integral gives:

$$\int_{0}^{2\pi }\cos(k_{1}x)dx=\frac{1}{k_{1}}\left[\sin(k_{1}\cdot 2\pi )-\sin(0)\right]$$ (12)

Sinusoidal functions have a period of 2π, so sin(2π·k)=0 for any integer *k*. Thus, this integral equals 0.

Since m,n≥1, n+m≥2 (ensuring n+m≠0). Let *k*_2_ = *n* + *m*; the second integral similarly evaluates to:

$$\int_{0}^{2\pi }\cos(k_{2}x)dx=\frac{1}{k_{2}}\left[\sin(k_{2}\cdot 2\pi )-\sin(0)\right]=0$$ (13)

Substituting these results back into the original equation confirms:

$$\int_{0}^{2\pi }\sin(nx)\sin(mx)\;dx=\frac{1}{2}\left(0-0\right)=0$$ (14)

**2. Orthogonality of cosine functions.** We aim to prove the orthogonality of cosine functions with distinct frequencies:

$$\int_{0}^{2\pi }\cos(nx)\cos(mx)\;dx=0$$ (15)

where $m,n\in {\mathbb{N}}^{+}$ (positive integers) and m≠n.

This derivation follows a logic analogous to that of the sine function orthogonality, starting with the trigonometric *product-to-sum identity* for cosines:

$$\cos A\cos B=\frac{1}{2}\left[\cos(A-B)+\cos(A+B)\right]$$ (16)

Substituting *A* = *nx* and *B* = *mx*, integrating over [0,2π], and applying the same logic for cosine integrals (each evaluates to 0 due to the periodicity of sine) yields:

$$\int_{0}^{2\pi }\cos(nx)\cos(mx)\;dx=\frac{1}{2}\left(0+0\right)=0$$ (17)

### 3.2 Orthogonal position representations

To balance the two core requirements of positional encoding in neural machine translation (NMT)—long-sequence extrapolation and position distinguishability—the Orthogonal Position Representation (OPR) method is built on a sinusoidal function framework, with targeted optimizations leveraging the orthogonality of trigonometric functions. We retain the sinusoidal function as OPR’s core structure, primarily due to its inherent periodicity—a property that overcomes the key limitation of learnable positional encodings. Learnable encodings are confined to sequence lengths encountered during training and fail to generate valid positional information for unseen longer sequences (e.g., document-level or long-sentence translation in practical NMT) without retraining. In contrast, the periodicity of sine and cosine functions enables natural extrapolation to longer sequences: even when sequence length exceeds the training maximum, their periodic pattern provides consistent positional cues, ensuring stable model performance on ultra-long inputs.

To address the insufficient distinguishability of vanilla sinusoidal encoding, we redesign the encoding structure using the orthogonality of sine and cosine functions. Vanilla sinusoidal encoding injects positional information via variable-frequency sine and cosine functions, but its periodicity causes overlapping encoding values for distant positions—weakening the Transformer’s ability to identify token order, which harms NMT as long-range dependencies directly affect translation coherence and accuracy. As formally proven in Sect 3.1, the orthogonality of sine and cosine functions within the interval [0,2π] (i.e., the integral of their product over this interval equals zero) guarantees non-redundant, linearly independent encoding vectors for any two positions—even distant ones—eliminating overlapping encodings and enhancing position distinguishability.

**Fig 3:**
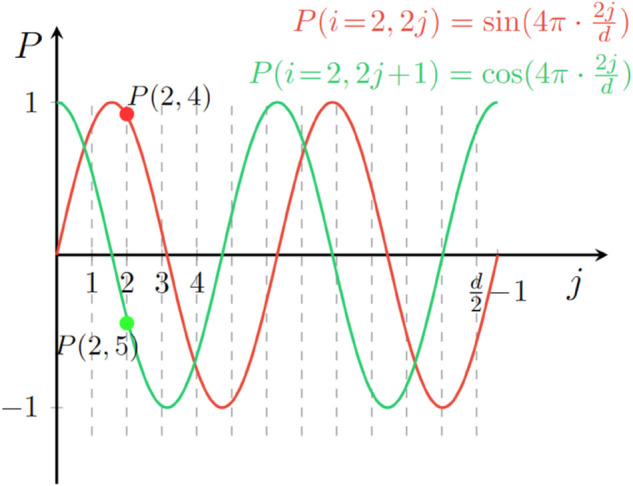
An example of the second positional encoding with *k* = 1. sin(2x) and cos(2x) are divided into $\frac{d}{2}$ parts respectively and spliced into *d* parts as the second positional encoding.

As established in the preceding section, the orthogonality of sine and cosine functions over [0,2π] is characterized by the following lemmas (consistent with Eqs (8) and (15)):

$$\int_{0}^{2\pi }\sin(nx)\sin(mx)dx=0$$ (18)

$$\int_{0}^{2\pi }\cos(nx)\cos(mx)dx=0$$ (19)

where *m*, *n* (m≠n) are numbers greater than or equal to 1. In this work, *m* and *n* denote two positions. Formally, we divide the period of the sine and cosine functions (from 0 to 2π) into $\frac{d}{2}$ parts, then the positional encoding can be presented as:

$$P\left(pos,2j\right)=\sin(pos\cdot 2\pi \cdot \frac{2j}{d})$$ (20)

$$P\left(pos,2j\;+\;1\right)=\cos(pos\cdot 2\pi \cdot \frac{2j}{d})$$ (21)

where *p*_*pos*_ represents the encoded absolute position, 2*j* and 2j+1 denote the even and odd dimensions of the position encoding, respectively. *d* is the dimensionality of the absolute position encoding, and j∈[0,d/2) represents the index of the dimension. In this way, Eq (20) and Eq (21) are two orthogonal functions, which can be regarded as the approximations of Eq (18) and Eq (19), respectively. Take *pos* = 2 for an instance (see Fig 3).

The position encodings generated by this method have an inner product of zero between the encodings of any two positions, i.e., $[p_{m},p_{n}]=0$. However, completely orthogonal vectors are difficult to learn for Transformer. Another problem is that they cannot capture the underlying relationship in neighboring positions. To address this drawback, we introduce a coefficient *k* to control how different orthogonal positions are. The main goal is to keep high similarity among relative positions and make the position representations be orthogonal among distant positions. We redefine the OPR as follows:

$$P\left(pos,2j\right)=\sin(pos\cdot \frac{2\pi }{k}\cdot \frac{2j}{d})$$ (22)

$$P\left(pos,2j\;+\;1\right)=\cos(pos\cdot \frac{2\pi }{k}\cdot \frac{2j}{d})$$ (23)

where *d* is the dimension size of the positional encoding, *k* is a coefficient larger than one. Unlike APE, each position and each dimension of the orthogonal position encoding corresponds to a sinusoid with a different frequency. Besides, the larger the *k* value, the larger the frequency will be.

### 3.3 Position-wise cosine similarity

For traditional APE, the encoding formula (applicable to both even and odd dimensions) is given by:

$$p_{\text{APE}}(i,j)=\sin\left(i\cdot 10000^{-2j/d}\right)\;\text{or}\;\cos\left(i\cdot 10000^{-2j/d}\right)$$ (24)

The key parameter is the frequency term $\omega_{\text{APE}}(j)=10000^{-2j/d}$, whose value decreases sharply with increasing dimension *j*—a critical factor limiting the value range of APE in high dimensions. Taking the high dimension *j* = 256 (with *d* = 512) as an example, the frequency term calculates to:

$$\omega_{\text{APE}}(256)=10000^{-2\times 256/512}=10000^{-1}=10^{-4}$$ (25)

For positions i∈[1,500], the input to the trigonometric function, $x_{\text{APE}}=\omega_{\text{APE}}(j)\cdot i$, ranges as:

$$x_{\text{APE}}\in [10^{-4}\times 1,10^{-4}\times 500]=[10^{-4},0.05]$$ (26)

Since $x_{\text{APE}}\ll 1$, we use small-angle approximations sin(x)≈x and $\cos(x)\approx 1-\frac{x^{2}}{2}$, leading to extremely narrow value ranges for APE:

- Sine terms (even dimensions): $p_{\text{APE}}(i,j)\approx x_{\text{APE}}\in [10^{-4},0.05]$ with a range width of only 0.0499;

- Cosine terms (odd dimensions): $p_{\text{APE}}(i,j)\approx 1-\frac{x_{\text{APE}}^{2}}{2}\in [0.99875,0.9999995]$ with a range width of merely 0.0012495.

**Fig 4:**
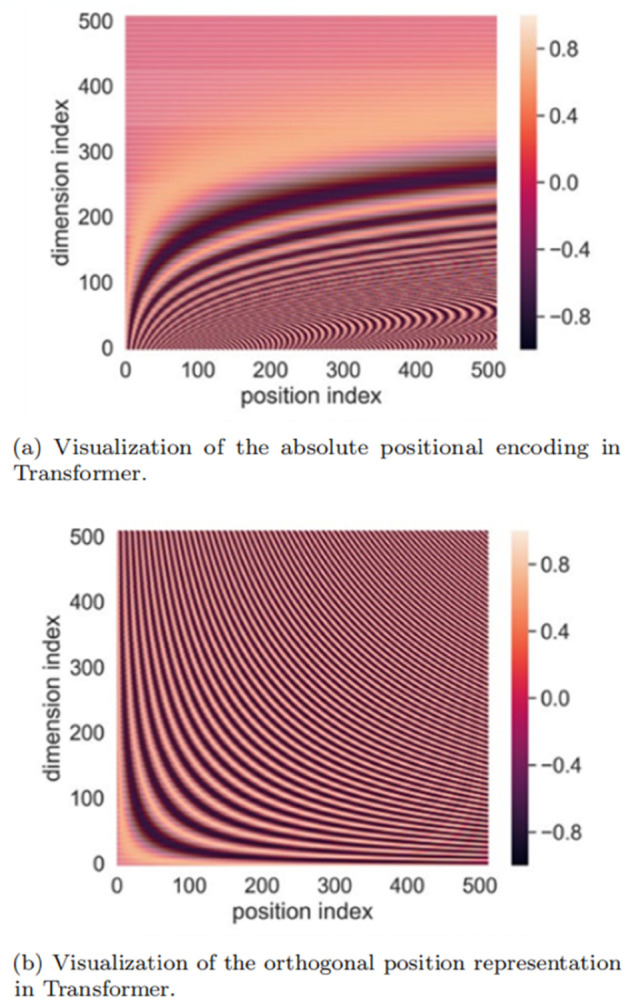
Visualization of two absolute positional encoding methods.

Thus, APE values in high dimensions are compressed into minimal intervals, resulting in negligible differences across positions (see Fig 4).

For the proposed OPR, the encoding formula (applicable to both even and odd dimensions) is:

$$p_{\text{OPR}}(i,j)=\sin\left(i\cdot 2\pi \cdot \frac{2j}{d}\right)\;\text{or}\;\cos\left(i\cdot 2\pi \cdot \frac{2j}{d}\right)$$ (27)

The frequency term here is $\omega_{\text{OPR}}(j)=2\pi \;\cdot \;\frac{2j}{d}$, which approaches 2π for large *j*—enabling OPR to cover full periodic ranges in high dimensions. For *j* = 256 (with *d* = 512):

$$\omega_{\text{OPR}}(256)=2\pi \cdot \frac{2\times 256}{512}=2\pi$$ (28)

For positions i∈[1,500], the input $x_{\text{OPR}}=\omega_{\text{OPR}}(j)\cdot i$ spans:

$$x_{\text{OPR}}\in [2\pi \times 1,2\pi \times 500]=[2\pi ,1000\pi ]$$ (29)

This range covers 1 to 500 full periods of the sine/cosine functions (period = 2π) (see Fig 4). Since sin(x) and cos(x) uniformly span [–1,1] over any integer number of periods, OPR values exhibit:

- Sine terms (even dimensions): $p_{\text{OPR}}(i,j)=\sin(x_{\text{OPR}})\in [-1,1]$ with a range width of 2;

- Cosine terms (odd dimensions): $p_{\text{OPR}}(i,j)=\cos(x_{\text{OPR}})\in [-1,1]$ with the same range width of 2.

**Fig 5:**
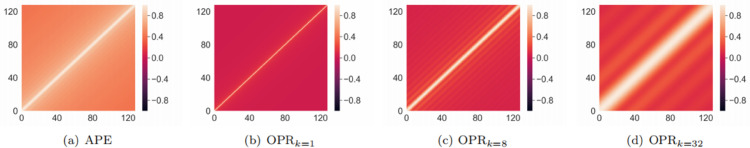
Visualization of position-wise cosine similarity of different position embeddings. Lighter denotes the higher similarity.

The narrow range of APE values directly translates to higher positional similarity, as visualized in Fig 5, which plots the visualization of position-wise cosine similarity of APE and the proposed OPR within different *k*. The point at (*n*,*m*) indicates the cosine similarity between the *n*-th positional encoding and the *m*-th positional encoding. We observe that APE always yields a higher cosine similarity than OPR. In the proposed OPR method, the cosine similarity of two different positions is zero when *k* = 1. When the value of *k* increases, the similar range of the current position to other positions is expended.

**Fig 6:**
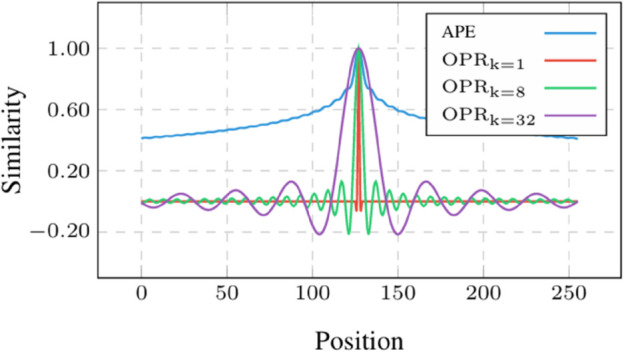
The similarity between the 128th positional encoding and others.

In this work, we set the value of *k* to 8. We hope that the positional encodings with a certain range are similar. It may help the model learn relative position information. At the same time, the long-distance positional encodings are different. By concealing long-distance information, the model pays more attention to a certain range of positional relationships. For example, the cosine similarity between the 128th positional encodings and other positional encodings is shown in Fig 6. We can see that when *k* is larger, the 128th positional encoding is easier to distinguish from the farther positional encoding and possess a potential connection with the short-distance positional encoding.

## 4 Experiments and results

We evaluated OPR on four widely used machine translation (MT) benchmarks: WMT’14 English-German (En-De), WMT’16 English-Romanian (En-Ro), IWSLT’14 English-German (En-De), and IWSLT’17 English-French (En-Fr). For comparative analysis, we performed experiments against two baseline positional encoding methods: Absolute Positional Encoding (APE) and Learned Positional Encoding (LPE).

### 4.1 Data

We followed the setup of the previous work [1,37].For the WMT’14 En-De task, the training data consisted of 4.5M sentence pairs. All sentences were jointly byte pair encoded [38] with 32K merge operations using a shared vocabulary. For the WMT’16 En-Ro task, the training data consisted of approximately 610K tokenized sentences. The same operations as those used in the WMT’14 En-De task are applied to obtain a vocabulary of the same size. The training set of the IWSLT’14 En-De task contains 160k sentence pairs, while the training set of the IWSLT’17 En-Fr task contains 282k sentence pairs. After tokenization using Moses, the Byte Pair Encoding (BPE) method with a vocabulary size of 10K operations is applied to perform finer-grained segmentation of the parallel sentence pairs.

Additionally, for the WMT’14 En-De task, we use the newstest2013 news translation test set as the validation set and the newstest2014 news translation test set as the final test set. For the WMT’16 En-Ro task, we use the newstest2014 news translation test set as the validation set and the newstest2016 news translation test set as the final test set. For the IWSLT’14 En-De task and the IWSLT’17 En-Fr task, we use the validation and test sets provided with the dataset. Furthermore, the same preprocessing method applied to the training set is also applied to the test and validation sets.The data situation for all tasks is shown in Table 1.

**Table 1:** The dataset situation for different tasks.

	WMT’14 En-De	WMT’16 En-Ro	IWSLT’14 En-De	IWSLT’17 En-Fr
Train Set	4.5M	610K	160K	282K
Valid Set	3000	1999	7283	890
Test Set	3003	1999	6750	2614
Vocab Size	32K	32K	10K	10K

### 4.2 Model settings

We used the Fairseq [37] codebase and trained models across four standard parameter configurations: small, base, big, and deep [1,39]. These configurations are widely adopted in the literature, having been extensively validated as effective baselines for NMT tasks. Their common usage ensures fair comparability with existing methods, as they provide a well-established reference point for evaluating new techniques like OPR. Our choice of these configurations is further supported by their consistent performance in preliminary validation, where they demonstrated stable training dynamics and competitive baseline results—findings that are reinforced in our main experiments (see Sect 4.3), where OPR’s improvements are observed across all these settings.

For training, we used Adam optimizer with $\beta_{1}=0.9$, $\beta_{2}=0.997$, and $\epsilon =10^{-8}$. Across all four model configurations, we adopted the inverse square root learning rate schedule with 4K warmup steps, a setup that aligns with standard practices for these architectures and has been shown to facilitate robust convergence.

For base and deep models, we used 6 encoder layers and 6/24 decoder layers. The hidden layer size of self-attention was 512, and the size of feed-forward inner-layer was 2,048. We used 8 heads for attention and set the dropout rate to 0.1 for base models and 0.3 for deep models. For small models, the hidden layer size of self-attention was 512, and the size of feed-forward inner-layer was 1,204. We used 8 heads for attention and set the dropout rate to 0.3. For big models, the hidden layer size of self-attention was 1,024, and the size of feed-forward inner-layer was 4,096. We used 16 heads for attention and set the dropout rate to 0.3. For our OPR method presented in Sect 3.2, we set *k* = 8 on all tasks. For the RPR method presented in Sect 2.2, we set max_relative_length=8 on all tasks. For evaluation, the beam width was 5 and the length penalty was 1.0 for all tasks.

We batched sentence pairs based on approximate sentence length. For the base and small models, we limited the number of input and output tokens per batch to 4,096 per GPU. The difference between the two models is that the base model uses 8 GPUs, while the small model uses 1 GPU. For the deep and big models, the token limit was set to 2,048 tokens per GPU, with 8 GPUs used and gradient accumulation performed every 8 steps. The parameter configuration of different models is shown in Table 2.

**Table 2:** The parameter configuration of different models.

	Small	Base	Big	Deep
$\beta_{1}$	0.9	0.9	0.9	0.9
$\beta_{2}$	0.997	0.997	0.997	0.997
*ε*	10^−8^	10^−8^	10^−8^	10^−8^
Encoder layer	6	6	6	24
Decoder layer	6	6	6	6
Model Size	512	512	1024	512
FFN Size	1024	2048	4096	2048
Heads	8	8	16	8
Dropout	0.3	0.1	0.3	0.3
Label Smoothing	0.1	0.1	0.1	0.1
Warmup Steps	4K	4K	4K	4K
Length Penalty	1.0	1.0	1.0	1.0
Beam Size	5	5	5	5

### 4.3 Results

Table 3 summarizes the results of different model capacities on four tasks. In all four tasks, regardless of whether relative position encoding is used or not, the learnable position encoding yields the worst performance. When relative position encoding is not used, compared to the APE method, the OPR method shows improvements on the WMT’16 En-Ro, IWSLT’14 En-De, and IWSLT’17 En-Fr tasks, with an average BLEU score increase of 0.09 and an average COMET score increase of 0.13. This demonstrates that the proposed OPR method is effective in improving machine translation performance.

**Table 3:** Results on the WMT’14 En-De and the WMT’16 En-Ro tasks with Base model and the IWSLT’14 En-De and the IWSLT’17 En-Fr tasks with Small model.

Models	Base				Small					
	WMT’14 En → De		WMT’16 En → Ro		IWSLT’14 En → De		IWSLT’17 En → Fr		Avg.En → X	
	COMET	BLEU	COMET	BLEU	COMET	BLEU	COMET	BLEU	COMET	BLEU
APE	82.48	25.72	79.13	32.22	76.00	28.10	79.98	35.76	79.40	30.45
OPR	82.25	25.59	80.32	33.68	76.21	28.21	79.34	34.69	79.53	30.54
LPE	82.08	25.27	78.65	31.65	75.05	27.45	78.51	34.86	78.57	29.81
APE+RPR	82.72	26.33	**80.80**	33.81	76.60	28.71	80.45	36.68	80.14	31.38
OPR+RPR	**82.95**	**26.46**	80.69	**33.82**	**76.89**	**28.93**	80.62	**36.87**	**80.29**	**31.52**
LPE+RPR	82.19	26.27	80.42	33.78	76.63	28.34	**80.63**	36.65	79.97	31.26

At the same time, to verify the compatibility of the OPR method with relative position encoding methods, we introduce the relative position encoding into the standard Transformer model for a comparative experiment. The experimental results show that our proposed method leads to significant improvements over the baseline model across all four tasks, with an average BLEU score increase of 0.14 and an average COMET score increase of 0.15. This proves that our method can help the model capture richer positional information on top of existing relative position encoding methods.

In addition, we further validated our approach on both deep and wide models. For the deep model, we selected a 24-layer Transformer model as the baseline and conducted experiments. The results of this experiment are shown in Table 4. Our method outperforms the baseline by 0.12 COMET points and 0.05 BLEU points. For the deep model, we also introduced the relative position encoding method for comparison experiments, which further improves the BLEU score by 0.25 and the COMET score by 0.29. At this point, the performance gain has started to diminish. One reason for this phenomenon is that the relative position encoding method acts on the attention sub-layers in each layer of the model. In deep models, the relative position encoding can capture richer positional information, which plays a similar role to the method we proposed, increasing the model’s ability to capture relative positional information. As a result, the positional information provided by our proposed method is somewhat affected.

**Table 4:** Results on the WMT’14 En-De task with Deep and Big model.

Models	Deep		Big	
	COMET	BLEU	COMET	BLEU
APE	83.63	27.07	83.80	27.27
OPR	83.75	27.12	**84.32**	27.73
LPE	82.13	25.63	83.51	27.24
APE+RPR	83.51	27.09	84.05	27.77
OPR+RPR	**83.80**	**27.34**	84.08	**27.84**
LPE+RPR	83.47	27.13	84.04	27.68

For the wide model, we selected the Transformer-Big model with a hidden layer size of 1024. As shown in the table, our method outperforms the baseline by 0.52 COMET points and 0.46 BLEU points. On top of the relative position encoding method, the performance is 0.03 COMET points and 0.07 BLEU points.

From Tables 3 and 4, we can observe clear differences in the performance gains of OPR relative to APE across different model complexity configurations. Under Base and Small model settings, which are characterized by smaller hidden layer dimensions and fewer parameters, OPR achieves relatively modest improvements: the average COMET score increases by only 0.13, and the average BLEU score by 0.09. In contrast, under the Big model configuration—consistent with the Transformer-Big architecture and featuring a 1024-dimensional hidden layer—OPR delivers significantly more prominent gains, with a COMET score increase of 0.52 and a BLEU score increase of 0.46. This pattern aligns with the core design principle of OPR detailed in Sect 3: OPR leverages an orthogonal fixed-dimension structure to maximize the discriminability of positional representations, and this design exhibits stronger advantages in high-dimensional spaces. Here, the larger hidden layer dimension provides sufficient “dimensional space” for OPR’s orthogonal vectors to fully exert their positional discrimination ability.

Overall, the OPR method does not introduce any additional parameters or computational overhead. It delivers considerable performance gains across lightweight models, deep models, and wide models—with particularly outstanding performance on resource-rich tasks. Meanwhile, this method can be synergistically used with the RPR method, leading to better results.

### 4.4 Ablation study

To validate the impact of hyperparameter *k* on the proposed similarity-based orthogonal position representation (OPR) method, we conducted ablation experiments on the WMT’14 English-German (En-De) task. The experiments cover two model configurations (Base and Big) and two settings (OPR used alone, and OPR combined with relative positional encoding (OPR+RPR)), with *k* values set to 1, 4, 8, 16, and 32. Detailed results are presented in Table 5.

**Table 5:** Results of OPR on WMT’14 En-De with different hyperparameter *k* values.

Models	Hyperparameter *k*	Base		Big	
		COMET	BLEU	COMET	BLEU
OPR	1	81.98	25.06	83.69	27.33
	4	82.13	25.52	83.84	27.56
	8	**82.25**	**25.59**	**84.32**	**27.73**
	16	82.12	25.49	83.95	27.70
	32	82.03	25.44	83.88	27.60
OPR+RPR	1	82.67	26.17	83.76	27.43
	4	82.85	**26.46**	83.92	27.62
	8	**82.95**	**26.46**	**84.08**	**27.84**
	16	82.81	26.36	83.98	27.73
	32	82.75	26.30	84.01	27.71

For the OPR method used alone, clear performance trends emerge with varying *k* across both Base and Big model configurations. In the Base model, the lowest performance is observed when *k* = 1 : the COMET score is 81.98 and the BLEU score is 25.06. As *k* increases to 8, performance reaches its peak—COMET rises to 82.25 and BLEU to 25.59. Further increasing *k* to 16 or 32 leads to a gradual decline in both metrics: at *k* = 32, COMET drops to 82.03 and BLEU to 25.44. The Big model follows a nearly identical pattern: *k* = 1 yields the lowest scores (COMET 83.69, BLEU 27.33), *k* = 8 achieves the best performance (COMET 84.32, BLEU 27.73), and larger *k* values (16, 32) result in slight performance degradation.

When OPR is combined with RPR (OPR+RPR), the optimal *k* remains consistent, though the performance range is overall higher than OPR alone. In the Base model, *k* = 4 and *k* = 8 tie for the highest BLEU score (26.46), while *k* = 8 achieves the highest COMET score (82.95)—outperforming *k* = 1 (COMET 82.67, BLEU 26.17) and *k* = 32 (COMET 82.75, BLEU 26.30). In the Big model, *k* = 8 again becomes the optimal choice, with COMET 84.08 and BLEU 27.84—surpassing all other *k* values, including *k* = 1 (COMET 83.76, BLEU 27.43) and *k* = 32 (COMET 84.01, BLEU 27.71).

The underlying reason for these trends lies in the role of *k* in shaping positional encoding similarity. When *k* = 1, the cosine similarity between any two positional encodings is zero, leading to fully orthogonal representations. This complete orthogonality severs implicit connections between adjacent positions, making it difficult for the Transformer to capture relative positional relationships and thus limiting performance. As *k* increases to 8, short-distance positions exhibit appropriate similarity while long-distance positions remain approximately orthogonal—this balance provides the model with sufficient cues to learn both absolute positions and latent relative positional information. When *k* exceeds 8, excessive similarity between short-distance positions begins to blur positional distinctions, weakening the model’s ability to differentiate nearby tokens and causing performance to decline.

In summary, the ablation experiments confirm that *k* = 8 is the optimal choice for both Base and Big models, and for both OPR alone and OPR+RPR settings. This value strikes a critical balance between preserving positional differentiation and enabling the capture of relative positional relationships, thereby maximizing translation performance.

### 4.5 Statistical tests

To confirm whether the performance advantage of the OPR method over the APE method is statistically significant, we pre-specified the statistical analysis framework before conducting experiments. We set the significance level *α* to 0.05 upfront—a value widely recognized as a conventional standard in neural machine translation (NMT) research [40–42]. This threshold effectively controls the Type I error (false positive error) probability while balancing the rigor of validating true performance differences and the avoidance of overly conservative inference that might overlook meaningful improvements. For hypothesis testing, we adopted a two-tailed paired t-test, the standard method for comparing paired samples. In this study, the five independent training runs of APE and OPR shared identical experimental configurations, with only the position encoding module differing; this qualifies them as paired data, and the method accurately captures differences between the two groups while accounting for shared experimental conditions. We defined the null hypothesis *H*_0_ as “the average BLEU scores of the APE and OPR methods are equal,” meaning there is no inherent performance difference between the two methods. The alternative hypothesis *H*_1_ was defined as “the average BLEU scores of the APE and OPR methods differ,” aiming to verify whether OPR possesses a significant performance advantage.

To ensure the validity of the aforementioned pre-specified statistical framework, we selected the WMT’14 English-German (En-De) task as the benchmark and strictly controlled experimental variables. The WMT’14 En-De task is a well-recognized benchmark in NMT research, featuring a large-scale parallel corpus with over 4.5 million sentence pairs. The abundant data volume reduces the impact of data sparsity on experimental results, ensuring that observed performance differences are more likely to stem from the design of the position encoding module rather than insufficient data. All experiments strictly followed the “Big model” parameter settings, with the position encoding module being the only variable difference between APE and OPR. This design eliminates confounding factors such as model size and training strategy, ensuring a fair comparison. The choice of “Big model” parameter settings is based on two key reasons: first, a larger parameter scale enhances the model’s ability to capture complex linguistic dependencies, which is crucial for the En-De translation task given the significant syntactic and structural differences between English and German; second, compared to smaller models, big models exhibit higher training stability and are less prone to extreme performance fluctuations caused by random initialization or minor training perturbations—this property is essential for conducting five independent training runs and obtaining reliable, comparable data.

Table 6 summarizes the BLEU scores from the five independent training runs of APE and OPR, along with key descriptive statistics (mean, standard deviation) and the results of the pre-specified t-test. For APE, the BLEU scores across the five training runs were 27.27, 27.21, 27.30, 27.26, and 27.23, with an average of 27.25 and a standard deviation of 0.04—this result indicates stable but relatively low overall performance. In contrast, OPR outperformed APE in every training run, achieving BLEU scores of 27.73, 27.65, 27.71, 27.74, and 27.68. This enabled OPR to reach an average BLEU score of 27.70 with a standard deviation of 0.04, representing an average absolute improvement of 0.45 BLEU points over APE. This not only demonstrates better overall performance but also maintains training stability comparable to that of APE.

**Table 6:** BLEU scores of 5 independent runs and statistical test results for APE vs. OPR on WMT’14 En-De task with Big model.

Method	Run 1	Run 2	Run 3	Run 4	Run 5	Mean ± Std	p-value
APE	27.27	27.21	27.30	27.26	27.23	27.25 ± 0.04	<0.05
OPR	27.73	27.65	27.71	27.74	27.68	27.70 ± 0.04	-

Critical to validating this performance gap, the paired t-test yielded a p-value <0.05. Since this p-value is lower than our pre-specified significance level, we can clearly reject the null hypothesis *H*_0_. This result confirms that the performance improvement of OPR over APE does not stem from random fluctuations during training or initialization, but rather is a statistically significant outcome. The root cause lies in the enhanced ability of OPR to model positional information—specifically, its optimized orthogonality design for distant tokens, which effectively addresses the inherent limitation of insufficient positional distinguishability in APE.

### 4.6 Analysis

#### 4.6.1 Analysis on different sentence lengths.

In this section, we analyze the performance of the proposed similarity-based absolute position encoding method on the WMT14 En-De task. We conducted comparative experiments on the translation results of two absolute position encoding methods at different sentence lengths, as shown in Table 7.

**Table 7:** Experimental results on sentences of different lengths.

Models	0-19 (1339)		20-39 (1396)		40-59 (254)		60-91 (14)	
	COMET	BLEU	COMET	BLEU	COMET	BLEU	COMET	BLEU
APE	84.17	24.79	81.81	25.85	77.69	25.97	75.29	32.84
OPR	83.91	25.07	81.66	25.51	77.26	25.73	74.02	**36.59**
APE+RPR	84.48	25.51	82.43	26.60	78.20	26.58	75.21	35.17
OPR+RPR	**84.71**	**25.76**	**82.60**	**26.62**	**78.91**	**26.74**	**76.94**	35.06

We split the parallel sentence pairs in the test set into four groups based on the number of English words: 0-19, 20-39, 40-59, and 60-91, containing 1339, 1396, 254, and 14 sentence pairs, respectively. From the experimental results, we observe that on the standard Transformer model, the proposed OPR method achieves comparable translation quality to the baseline APE method in the shorter sentence length ranges. However, it performs better on longer sentences. After incorporating the relative position encoding (RPR) method, we find that the proposed OPR method outperforms the APE method in translation quality for sentence lengths within the range of 0-59. However, it performs worse than APE on longer sentences.

We attribute this to the fact that the relative position encoding method itself has a better relative position modeling mechanism, which captures more rich relative position information. Additionally, in our experiment, we set the value of *k* to 8, which led the method to focus more on modeling short-range relationships. For longer sentences, it would be beneficial to choose a larger *k* value to model long-distance relationships more effectively.

#### 4.6.2 Perplexity analysis.

We further evaluated the performance of the proposed OPR method during the model training phase by comparing its perplexity (PPL) with that of the baseline APE method. Perplexity serves as a critical metric for assessing how well a model predicts a sequence of tokens, with lower values indicating better predictive performance.

**Fig 7:**
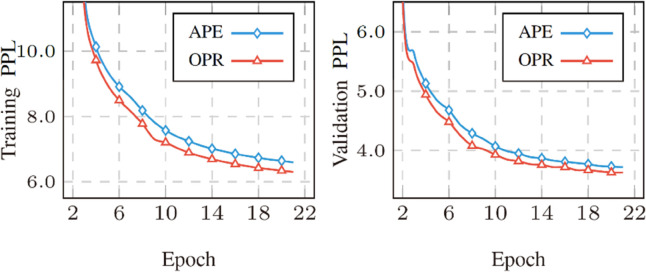
Perplexity comparison during training.

As illustrated in Fig 7, the OPR method consistently achieves lower perplexity than the sine-cosine-based APE method at every training checkpoint. This trend holds for both the training set and the validation set: across all stages of training, OPR’s PPL remains below that of APE. This result demonstrates that the proposed OPR method enhances the model’s ability to learn and predict token sequences throughout the entire training process, laying a more solid foundation for improved translation performance in the inference phase.

#### 4.6.3 Complexity analysis.

This section analyzes the parameter complexity and computational complexity of two absolute positional encoding methods: the conventional sine-cosine absolute positional encoding (APE) and the proposed similarity-based absolute positional encoding (OPR).

For a Transformer model with a maximum sequence length of *n* and a hidden dimension of *d*, both positional encoding methods generate positional representations that align with the model’s input sequence length and hidden dimension. From the perspective of parameter complexity: neither APE nor OPR introduces additional trainable parameters. APE computes positional vectors directly via fixed sine-cosine functions, while OPR constructs orthogonal positional representations based on a predefined similarity mechanism—both methods avoid parameter storage overhead, resulting in a parameter complexity of *O*(*nd*) (consistent with the dimension of the positional vectors themselves, which is inherent to the model’s input requirements rather than method-specific extra parameters).

In terms of computational complexity: The core operation of APE involves calculating sine and cosine values for each position and dimension, which requires *O*(*nd*) floating-point operations. For OPR, the orthogonal positional representation is generated through matrix operations that scale linearly with the sequence length *n* and hidden dimension *d*, also resulting in a computational complexity of *O*(*nd*).

In summary, compared with the sine-cosine APE method used in the standard Transformer model, the proposed OPR method does not increase the model’s parameter count or computational overhead. This ensures that OPR can be seamlessly integrated into existing Transformer-based frameworks without compromising efficiency—an essential advantage for practical industrial applications and large-scale model training.

## 5 Limitations

While the Orthogonal Position Representation (OPR) method offers advantages in enhancing the distinguishability of distant position encodings, it does have some inherent drawbacks that may affect its adaptability in certain scenarios. One aspect worth noting is the method’s sensitivity to the coefficient *k*–a trait it shares with the Relative Position Representation (RPR) [11] method, where the performance is similarly reliant on appropriate parameter calibration for relative position biases. . Though *k* serves the purpose of balancing orthogonality and the correlation between adjacent positions, there is currently no established theoretical basis for determining its optimal value across various tasks or datasets. In practice, identifying a suitable *k* often requires a certain degree of empirical tuning, which may introduce additional computational efforts and, in cases of less precise calibration, could lead to suboptimal performance to some extent. Another point is that while the periodicity of sinusoidal functions provides OPR with a certain capacity for extrapolating to longer sequences, this capability is somewhat constrained by the fixed frequency scaling based on *k* and dimension *d*. When dealing with sequences significantly longer than those encountered during training, the frequency differences between distant positions might become less pronounced, which could moderately reduce the orthogonality of their encodings and thus affect their distinguishability.

## 6 Conclusion

In this paper, we design an orthogonal fixed-dimension positional representation to discriminate positions more easily for Transformer. This method is especially effective for neural machine translation (NMT) tasks, where the precise understanding of word positions within a sequence is crucial for high translation quality. Through experiments on multiple datasets (WMT’14 En-De, WMT’16 En-Ro, IWSLT’14 En-De, and IWSLT’17 En-Fr), the OPR method showed consistent improvements. The results also demonstrated that the OPR method is compatible with existing models and can be used in conjunction with other techniques, like RPR, to further improve performance. Additionally, we provide insights into how the OPR method performs across sentences of different lengths. It is found that the method excels in translating longer sentences, especially when combined with RPR, indicating the potential effectiveness of the OPR method in document-level translation. In conclusion, the OPR method is a significant advancement in positional encoding for Transformer models, particularly for NMT tasks, and offers improved performance when combined with relative position encoding techniques. The paper contributes a new approach to positional information representation in deep learning models, offering both theoretical insights and practical improvements in translation systems.
